# Three Thousand Questions on Medical Subjects

**Published:** 1891-11

**Authors:** 


THREE THOUSAND QUESTIONS ON MEDICAL SUBJECTS. Arranged for self-examination with the proper references to standard works in which the correct replies will be found. Philadelphia: P. Blakiston, Son & Co., 1012 Walnut Street, 1891.

This little work is sufficiently described in its title. It contains a most excellent set of questions which for all practical purposes cover the whole field of medicine. At the end of each question is a number or a letter, which on consulting the key at the beginning of the book, will enable the student to find the proper answer in a standard work. These works consist of Blakistons' celebrated series of Quiz-compends, *Gray's Anatomy*, eleventh edition, and Gould's *New Medical Dectionary*, advertised in the pages of this journal at the beginning of the present year.

The Messrs. Blakiston desire us to announce that they will send this little book *free* to all medical students sending them TEN CENTS in stamps to cover cost of mailing.

We cordially commend the book to students and practitioners alike.

